# Age-associated changes in human tear proteome

**DOI:** 10.1186/s12014-019-9233-5

**Published:** 2019-03-30

**Authors:** Janika Nättinen, Antti Jylhä, Ulla Aapola, Petri Mäkinen, Roger Beuerman, Juhani Pietilä, Anu Vaajanen, Hannu Uusitalo

**Affiliations:** 1 SILK, Department of Ophthalmology, Faculty of Medicine and Health Technology, Tampere University, PL 100, 33014 Tampere, Finland; 2 Silmäasema Eye Hospital, Tampere, Finland; 3 Singapore Eye Research Institute, Singapore, Singapore; 4 Duke-NUS Medical School Ophthalmology and Visual Sciences Academic Clinical Program, Singapore, Singapore; 5 Tays Eye Centre, Tampere University Hospital, Tampere, Finland

**Keywords:** Aging, Mass spectrometry, Ocular surface, Proteomics, SWATH-MS, Tear fluid

## Abstract

**Background:**

Prevalence of many eye and ocular surface diseases increases with age. While the clinical characteristics and pathophysiologic mechanisms of these conditions are often either known or extensively studied, the effects of normal aging on tear film and ocular surface have not been as widely researched.

**Methods:**

In order to examine the effects of aging on tear fluid proteomics, tear fluid samples were collected preoperatively from 115 subjects undergoing strabismus or refractive surgery using glass microcapillary tubes. In addition to their refractive error or strabismus, the subjects did not have any other current, known eye diseases. The non-pooled samples were analysed using NanoLC-TripleTOF implementing a sequential window acquisition of all theoretical fragment ion spectra mass spectrometry, resulting in quantified data of 849 proteins.

**Results:**

According to correlation results, 17 tear proteins correlated significantly with increased age and many of these proteins were connected to inflammation, immune response and cell death. According to enrichment analysis, growth and survival of cells decreased while immune response and inflammation increased with aging. We also discovered several well-known, activated and inhibited upstream regulators, e.g. NF-κB, which has been previously connected to aging in numerous previous studies.

**Conclusions:**

Overall, the results show the common age-dependent alterations in tear fluid protein profile, which demonstrate similar age-associated alterations of biological functions previously shown in other tissue and sample types.

**Electronic supplementary material:**

The online version of this article (10.1186/s12014-019-9233-5) contains supplementary material, which is available to authorized users.

## Background

Older age is a major risk factor for various chronic eye diseases, such as age-related macular degeneration, glaucoma, dry eye and other ocular surface diseases [1, 2]. In the future, the number of patients with these conditions is likely to increase due to population aging but also, in the case of ocular surface diseases, due to increased use of digital displays and environmental factors such as poor air quality. Therefore, there is a need for better understanding of normal molecular aging-effects in the eye in order to tackle the growing ocular surface issues. Fortunately, in recent years, research of ocular surface and its molecular functions has advanced due to technological developments in diagnostic methods. Tear fluid, that is nourishing and lubricating the underlying eye, provides a non-invasive source for sensitive proteomic analyses by means of mass spectrometry to detect putative biomarkers of ocular surface health.

The normal clinical effects of aging in the eye are relatively well-known and affect all parts of the eye. In ocular surface of the eye, the tear fluid, consisting of lipid, aqueous and mucin layers, which are produced respectively by meibomian glands, lacrimal glands and conjunctival goblet cells, is known to be altered during aging in many ways. Increased age results in lowered tear film stability and lacrimal gland secretion [3–9]. Tear film composition is also altered [10], similar to meibomian gland-produced lipid profiles [7, 11], while tear evaporation rate is elevated [12]. Despite the various studies on the clinical changes, the normal molecular changes in aging eye are not yet fully understood. On molecular level, increased inflammation and dysregulation of innate immune response have been connected to normal aging [13, 14] as well as ocular surface conditions [15, 16] and hence it can be hypothesised that aging could affect these biological pathways in ocular surface as well.

It has been further hypothesised that ocular surface aging patterns between women and men are different as women are more likely to suffer from age-related ocular surface conditions, such as dry eye and Sjögren’s syndrome [2, 17, 18]. Some evidence suggests that for example the lipid layer, while thinner and more contaminated among all elder people, is more affected by age with elder women [8] resulting in higher tear evaporation rate [12]. One hypothesis to explain the differences between ages and sexes, are the shifting levels of sex hormones, which have been connected to meibomian and lacrimal gland functions [19], and which are more prominent among post-menopausal women. However, as ocular surface diseases are often multifactorial, the underlying causes are expected to vary and be far more complex.

In this study, we focused on tear film proteomics in particular, which we hoped to provide further insight into the normal changes in ocular surface during aging and possibly also provide further information on the differences between sexes. Previously, McGill et al. [10] studied specific tear proteins and their age-related changes and discovered that there was an age-associated decline in the expression levels of antimicrobial lysozyme, lactoferrin and IgA, while ceruloplasmin and IgG were increased. More recently, Micera et al. [20] implemented protein array data to evaluate age-associated changes in tear fluid and confirmed that several pro-inflammatory interleukins and other proteins were increased with age. Although various age-related diseases have been studied using tear fluid proteomics [21–23], any wider mass spectrometry discovery studies have not been performed on the tear fluid proteome changes during normal aging, since methods enabling this type of research have only been developed quite recently. We gathered tear fluid samples from normal, healthy subjects of all ages undergoing either strabismus or refractive surgery and implemented mass spectrometry methods to obtain quantified proteomics data on individual patients. Our aims were to identify those proteins, which are increasing or decreasing in their expression with increasing age and to see how sex of the patient affects these changes. We hypothesized that the statistically significant proteins would be connected to age-associated biological functions such as immune and inflammatory response. To our knowledge, this is the first proteomics study to implement mass spectrometry to examine the tear fluid differences among people of different ages.

## Methods

### Study population

Subjects in this study originate from two separate studies, which implemented these subjects as healthy control populations. The subjects of the first study underwent a strabismus surgery (n = 30) and the subjects in the second study a femtosecond laser in situ keratomileusis (FS-LASIK) (n = 85). In both cases, open-eye tears were collected from the lower conjunctival cul-de-sac with capillaries prior to any manipulation or anaesthesia of the eye, including surgery.

The subjects undergoing refractive surgery had a complete preoperative ophthalmologic examination, including biomicroscopy, measurement of corneal thickness and three-dimensional corneal topography (Allegro Oculyzer, Wavelight AG, Erlangen, Germany) prior to the surgery and they had to discontinue wearing soft contact lenses at least 1 week before testing. Any anterior or other pathology of the eye that might be a contraindication for the refractive surgery, including dry eye disease, lid infection, corneal pathology, any prior ocular surgery or recent ocular infection, was an exclusion criterion. Other additional exclusion criteria were similar to any other refractive surgery: age (under 18 years old) and pregnancy. The subjects undergoing strabismus surgery similarly had a preoperative ophthalmic examination including biomicroscopy, fluorescein staining, conjunctival redness and Schirmer’s test in order to identify any clinical pathologies, which would be an exclusion criterion. Similarly, no subjects aged under 18 years or pregnant were included.

### Tear collection and sample preparation

The tear fluid samples were taken before installation of any surgery-associated eye drops. Tear samples were collected into 2 or 3 μl glass microcapillary tubes and stored at − 80 °C until assessed.

Samples were flushed from capillaries with 0.5% sodium dodecyl sulphate (SDS) in 50 mM ammonium bicarbonate supplemented with protease inhibitor cocktail and total protein concentration of the tear samples was measured by DC protein assay (Bio-Rad laboratories Inc, Hercules, USA) using bovine serum albumin as a standard. Total protein concentration of 5 µg was considered as a limit for proteomic analyses. 15 samples did not have sufficient amount of protein in the samples.

For protein analysis, acetone-precipitated proteins were dissolved in 2% SDS in 0.05 M triethylammonium bicarbonate buffer (TEAB) and reduced by tris-(2-carboxyethyl)phosphine (TCEP) for 1 h at +60 °C. The reduced samples were transferred into 10–30 kDa molecular weight cut-off filters and flushed with 8 M urea in 0.05 M Tris–HCl (Thermo Fisher Scientific, Waltham, USA) to remove the excess reagent. Cysteine residue blocking was done by iodoacetamide (IAA) at room temperature in the dark. Alkylation was terminated by centrifugation and the samples were washed with urea and 0.05 M TEAB prior to digestion with trypsin (Sciex, Framingham, USA) for 16 h at + 37 °C at a trypsin-to-protein ratio of 1:25. Digests were eluted from filters with 0.05 M TEAB followed by 0.5 M NaCl and dried in a speed vacuum concentrator. Samples were reconstituted in 0.1% trifluoroacetic acid (TFA), cleaned and desalted with Pierce C18 tips (Thermo Fisher Scientific) according to manufacturer’s instructions. After clean up the samples were vacuum dried and stored in − 20 °C until reconstituted to loading solution (2% ACN, 0.1% FA) at equal concentrations. Unless otherwise stated all reagents were purchased from Sigma-Aldrich (St. Louis, MO, USA). The samples were analysed with NanoLC-TripleTOF instrumentation using Eksigent 425 NanoLC coupled to high speed TripleTOF™ 5600 + mass spectrometer (Sciex, Concord, Canada). The analysis was performed using sequential window acquisition of all theoretical mass spectra (SWATH-MS). Further detailed information of the methods and parameters of NanoLC and TripleTOF have been published in our previous papers [21, 24].

### Protein identification and quantification and SWATH-MS library creation and peak integration

For SWATH-MS analysis method, we created a relative protein quantification library, consisting of > 950 proteins. This library was created using tear samples of this study as well as two other clinical studies consisting of glaucoma patients and dry eye patients. Overall library consisted of 55 different subjects/samples and over 80 data-dependent acquisition (DDA) runs with same liquid chromatography (LC) gradient and instrument settings, which were used for SWATH-MS analyses. Library was created using Protein Pilot^®^ 4.5 (Sciex, Redwood City, USA) and all DDA runs MS/MS spectra were identified against UniProtKB/Swiss-Prot. Quantification was performed using PeakView^®^ and MarkerView^®^ (Sciex, Redwood City, USA). False discovery rate (FDR) of 1% was used in the library creation and only distinctive peptides were used in the quantification. Retention time calibration was done for all samples using 8 peptides from lysozyme and 5 peptides from albumin. Five transitions per peptide and 1–15 peptides were used for peak area calculations. All proteins with significant or interesting findings in the data analysis were subjected to manual inspection of peptides. This consisted of checking correct peak selection in the chromatogram (FDR 1%, 99% peptide confidence level), sufficient signal to noise ratio inspection (> 7) and chromatogram inspection in relation to library chromatogram. In addition, variation of replicate MS analyses results was calculated as means to all samples/protein. If manual inspection requirements were not fulfilled, peptides were eliminated from results processing. Results are presented as combination of protein specific peptides peak intensities from SWATH-MS measurement and referred to as protein expression.

### Data processing and statistical analysis

Log_2_-transformation and central tendency normalization were used to normalize the protein quantification data. Majority of the samples had two replicate MS analyses run and their variation was calculated by intraclass correlation (ICC package in R) and by permutation tests using Spearman’s rank correlation. The replicate MS analyses were combined by taking geometric means.

The correlations between age and relative quantification data were performed with Pearson’s product-moment correlation. Ingenuity Pathway Analysis (IPA^®^) was implemented to evaluate the enriched biological functions and diseases based on correlation results (included measurements were p-value as “Expr p-value” and Pearson’s Rho as “Expr Other”). Sex differences were tested using Wilcoxon rank sum test. Benjamini–Hochberg adjustment was applied to all p-values and only proteins with an adjusted p-value below threshold (alpha = 0.05) were considered statistically significant unless otherwise stated. All statistical analyses for the proteomics data were performed using R software version 3.4.3 (R Core Team, Vienna, Austria) and QIAGEN’s IPA^®^ (QIAGEN Redwood City, USA).

## Results

### Clinical patient data

The study consisted of 115 subjects. Tear samples of 30 subjects undergoing strabismus surgery and 85 undergoing refractive surgery were collected prior to their operations. None of the subjects had any other current, known eye diseases; however, four strabismus surgery subjects had previously undergone a cataract surgery. The data consisted of 61 females (13 strabismus and 48 refractive surgery subjects) and 54 males (17 strabismus and 37 refractive surgery subjects). The median age for subjects was 41 years [95% CI 38–43.9] and ranged from 18 to 83. The median age for females was 40 [95% CI 37–43.8] and for males 42 [95% CI 37.3–46.7]. The age was not significantly different between female and male groups was (p = 0.6) according to Wilcoxon signed-rank test.

### Proteomics data

We identified 30,358 peptides from 115 samples, including MS analysis replicates, corresponding to 660,966 identified spectra in an assembly of 1497 protein groups using FDR of 1.0%. We included 950 proteins with distinctive peptides to quantification library, of which 849 proteins had distinct peptide sequences with matching spectra to SWATH-MS analysis. These proteins were quantified in all samples. The quality checks performed with the MS replicate analyses suggested that the proteomic data was of good quality as the mean of intraclass correlation coefficient was 0.957 and performing permutation tests (Spearman’s correlation) resulted in 86.6% of p-values < 0.05.

### Age affects tear proteins associated with inflammation and immune response

Tear protein profiles of the analysed 115 subjects were used to evaluate the relationship between tear protein expression and age as well as sex differences. Age was significantly correlated with several well-known tear proteins, many of which were associated with biologically important functions such as cell death and inflammatory and immune response based on IPA and gene ontology (GO) databases (Table 1). The positively correlating proteins included: cytoplasmic actins (ACTB and ACTG1), albumin (ALB), annexin A1 (ANXA1), carcinoembryonic antigen-related cell adhesion molecule 7 (CEACAM7), neutrophil defensin 1 (DEFA1), gelsolin (GSN), neutrophil gelatinase-associated lipocalin (LCN2), profilin-1 (PFN1), retinoic acid receptor responder protein 1 (RARRES1), mammaglobin-A (SCGB2A2), serotransferrin (TF), proteins S100A8 and S100A9. Ubiquitin-like modifier-activating enzyme 1 (UBA1) and Golgi membrane protein 1 (GOLM1) correlated negatively with age. Further information on the expression level profiles of these proteins within different age groups can be found from Additional file 1, which also lists the associated median and mean protein expression values as well as interquartile ranges and pairwise t-test p-values for patients grouped by age (5 groups: < 30, 30–39, 40–49, 50–59, and ≥ 60). These results suggest that for majority of the statistically significant proteins, the largest changes take place among the aging subjects after the age of 60, although for some proteins, the changes in expression occur already among the 50–59 year-olds’ group.

**Table 1:** List of proteins correlating significantly with age and the associated biological functions

Uniprot	Full name	Symbol	R^a^	p^b^	Cell death	Cellular movement	Inflammatory/immune response	Viral infection
P80188	Neutrophil gelatinase-associated lipocalin	LCN2	0.36	0.011	x	x	x	x
P05109	Protein S100-A8	S100A8	0.40	0.004	x	x	x	x
P06702	Protein S100-A9	S100A9	0.36	0.013	x	x	x	x
P60709	Actin, cytoplasmic 1	ACTB^‡^	0.31	0.036	x	x	x	x
P02768	Serum albumin	ALB	0.35	0.013	x	x	x	x
P04083	Annexin A1	ANXA1	0.37	0.010	x	x	x	x
P07737	Profilin-1	PFN1	0.32	0.036	x	x	x	
P06396	Gelsolin	GSN	0.32	0.036	x	x		
P02787	Serotransferrin	TF	0.37	0.010	x		x	
P63261	Actin, cytoplasmic 2	ACTG1^‡^	0.31	0.037	x			
P22314	Ubiquitin-like modifier-activating enzyme 1	UBA1	− 0.32	0.036	x			
P59665	Neutrophil defensin 1	DEFA1	0.40	0.004		x	x	
P49788	Retinoic acid receptor responder protein 1	RARRES1	0.30	0.046		x		
Q8NBJ4	Golgi membrane protein 1	GOLM1	− 0.31	0.036				x
Q13296	Mammaglobin-A	SCGB2A2	0.34	0.023				
Q14002	Carcinoembryonic antigen-related cell adhesion molecule 7	CEACAM7^†^	0.32	0.036				

^a^Pearson’s product-moment correlation coefficient

^b^Benjamini-Hochberg-adjusted p-value

^†^Expression based on 1 peptide only

^‡^Proteins ACTB and ACTG1 are isoforms of the same protein

**Fig. 1:**
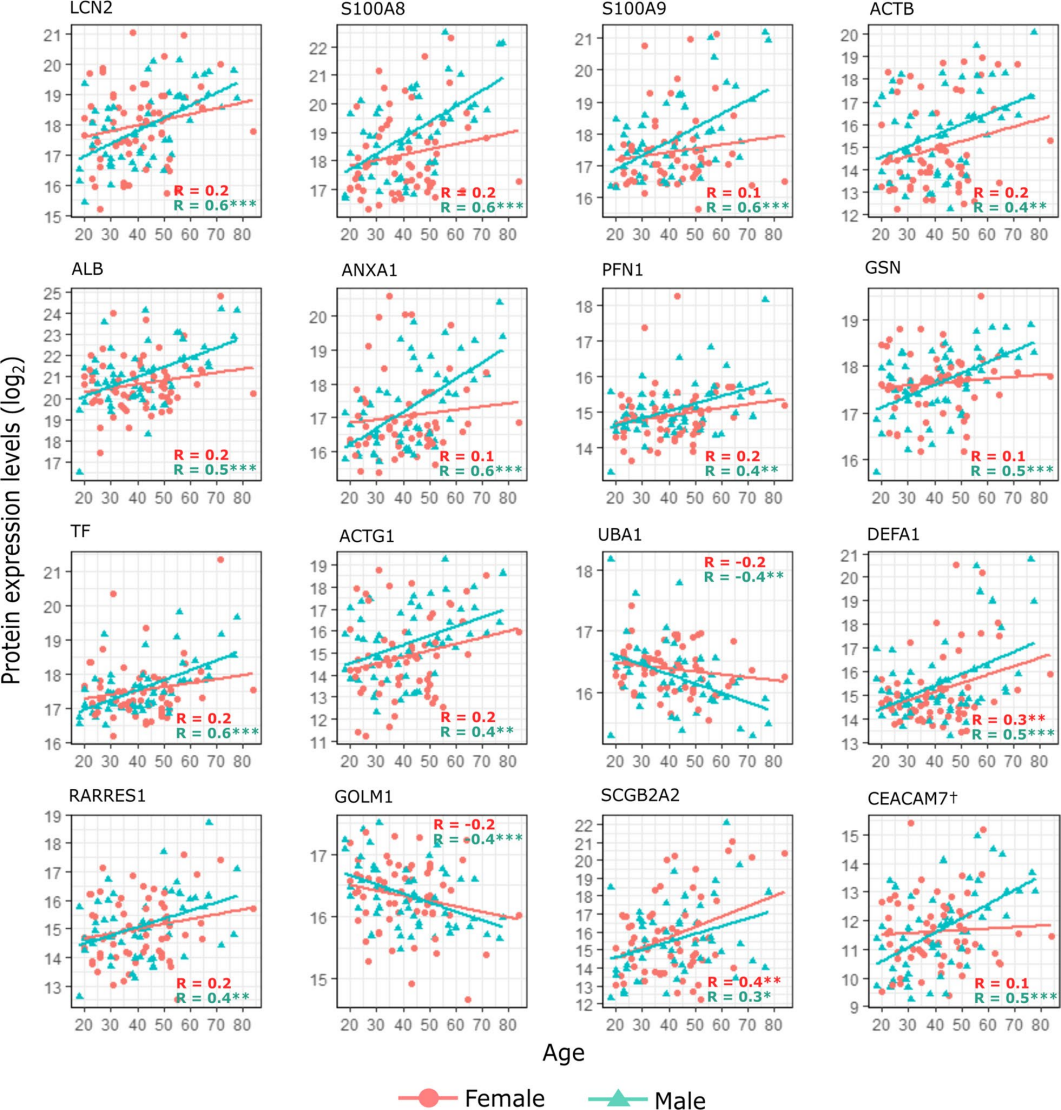
Proteins correlating significantly with age. Scatter plots display the statistically significant correlations (from Table 1) between protein expression levels (y-axis) and age (x-axis). Correlation results were also evaluated for males and females separately and, unless otherwise stated, only males’ expression levels correlated significantly with age for the displayed proteins. The colour and shape of the points signify males and females, which also have individual fitted lines and corresponding Rho (R) values displayed. ^†^CEACAM7 expression is based on only 1 unique peptide in the data. *p-value < 0.05, **p-value < 0.01, ***p-value < 0.001

Although sex alone was not significantly affecting protein expression levels in tears, some differences were observed when the age-affected proteins were analysed for male and female groups separately (Fig. 1). More specifically, the proteins were correlating with age similarly among both females and males, but the protein-age-correlations were in most cases statistically significant and more consistent with males. SCGB2A2 was the only protein, which appeared to have a higher correlation coefficient for female subjects, while e.g. S100A9, ANXA1, GSN and CEACAM7 had notably higher age-associated increase for males according to the linear regression lines shown in the figure.

**Fig. 2:**
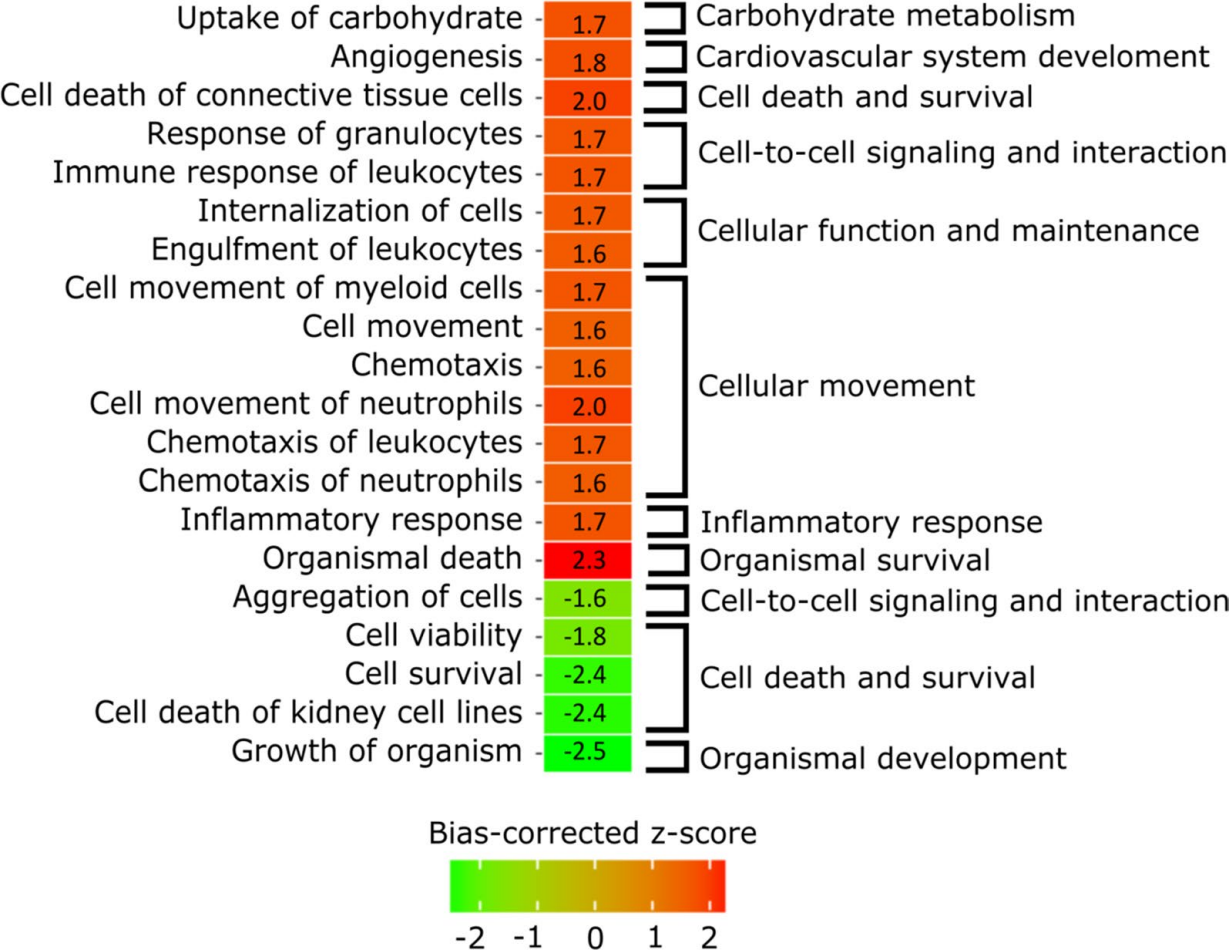
Enriched biological functions affected by aging. Heat map displays the general terms (excluding disease-specific terms such as cancer terms), which have either highly increased (red) or decreased (green) functions based on activation z-score (absolute bias-corrected z-score > 1.5, p-value < 0.05), as age is increased. On the left, the more specific terms are provided for each row and on the right, the specific terms are grouped under categories that are more general

The connections between tear proteomics and aging (for data including both sexes) were further examined by performing pathway analysis. The pathway analysis was performed on data with relaxed thresholds, more specifically using proteins with unadjusted p-value < 0.05. Additional file 1 includes further information about the results associated with this protein list. We focused on the enriched biological functions and upstream regulators. Some of the interesting biological function terms are visualized and grouped in Fig. 2 and more complete results can be found from Table 2. Increased biological functions include terms connected to immune response and more specifically migration of immune cells. In addition, there was evidence of increased inflammatory and cell death responses in the tears of elderly subjects. Cell viability and survival as well as growth of organism had negative z-scores, suggesting inhibition of these functions.

**Table 2:** Enriched diseases and biological functions filtered by activation z-score (absolute bias-corrected z-score > 1.5, p-value < 0.05) and ordered based on categories and sign of z-score

Categories	Diseases or functions annotation	Bias-corrected z-score	p
Carbohydrate metabolism	Uptake of carbohydrate	1.7	6.54E−04
Cardiovascular system development and function	Angiogenesis	1.8	4.98E−05
Cell death and survival	Cell death of connective tissue cells	2.0	6.98E−04
Cell-to-cell signaling and interaction	Response of granulocytes	1.7	2.06E−06
	Immune response of leukocytes	1.7	2.60E−03
Cellular function and maintenance	Internalization of cells	1.7	3.18E−03
	Engulfment of leukocytes	1.6	3.29E−04
Cellular movement	Cell movement of myeloid cells	1.7	1.23E−05
	Cell movement	1.6	4.22E−07
	Chemotaxis	1.6	1.15E−04
	Cell movement of neutrophils	2.0	5.20E−05
	Chemotaxis of leukocytes	1.7	5.48E−05
	Chemotaxis of neutrophils	1.6	1.26E−03
Inflammatory response	Inflammatory response	1.7	1.68E−05
Organismal survival	Organismal death	2.3	3.17E−05
Cell-to-cell signaling and interaction	Aggregation of cells	− 1.6	4.10E−05
Cell death and survival	Cell viability	− 1.8	3.63E−04
	Cell survival	− 2.4	1.90E−06
	Cell death of kidney cell lines	− 2.4	2.15E−03
Organismal development	Growth of organism	− 2.5	6.72E−05

**Fig. 3:**
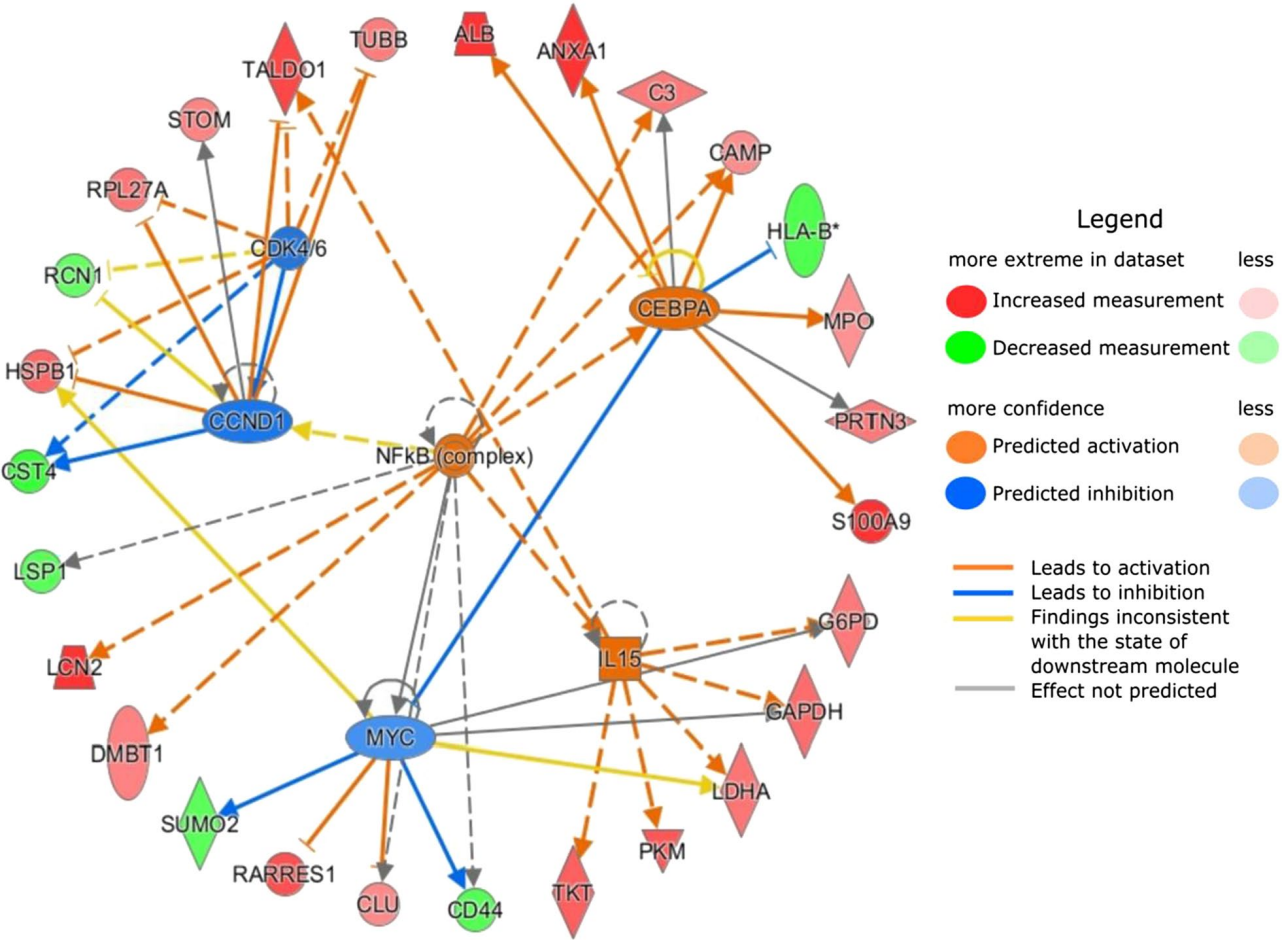
Upstream regulators associated with aging of the ocular surface. The graph displays the top 3 activated (in orange) and inhibited (in blue) upstream regulators based on the protein-age-correlation directions obtained. Proteins with increased (in red) and decreased (in green) measurements originate from the protein-age-correlation results, i.e. increased measurements suggest a positive correlation between the protein expression level and age, and decreased measurements a negative correlation

The upstream regulators, which could affect and cause the protein expression level changes we have observed in the data, are visualized in Fig. 3 and listed in Table 3. The three regulators with the highest activation (bias-corrected) z-scores were NF-κB complex, CCAAT/enhancer binding protein α (CEBPA) transcription factor and interleukin 15 (IL15) and the three molecules with the lowest activation z-scores were transcription regulators Myc proto-oncogene protein (MYC), cyclin D1 (CCND1) and cyclin-dependent kinase 4 and 6 (CDK4/6) group.

**Table 3:** Upstream regulator analysis filtered by p-value (p-value < 0.05) and ordered based on molecule type and sign of z-score

Upstream regulator	Molecule type	Bias-corrected z-score	p of overlap
NFκB (complex)	Complex	1.5	3.01E−04
IgG	Complex	0.5	3.65E−05
IL15	Cytokine	2.2	2.08E−05
IL13	Cytokine	0.9	1.63E−02
IFNG	Cytokine	0.1	7.23E−03
IL1B	Cytokine	0.1	7.27E−04
TNF	Cytokine	0.0	3.60E−09
MGEA5	Enzyme	0.2	3.28E−02
ERK1/2	Group	1.3	8.18E−05
Estrogen receptor	Group	0.8	4.54E−03
EGFR	Kinase	0.9	2.58E−03
EFNA4	Kinase	0.1	7.28E−05
ESRRA	Ligand-dependent nuclear receptor	1.5	3.00E−05
ESR1	Ligand-dependent nuclear receptor	1.0	2.70E−03
PCGEM1	Other	1.1	2.21E−06
EFNA1	Other	0.1	1.82E−04
CEBPA	Transcription regulator	2.2	3.28E−07
IL6	Cytokine	− 0.4	4.76E−03
TGM2	Enzyme	− 0.2	2.29E−02
CDK4/6	Group	− 1.4	1.66E−06
MAPK1	Kinase	− 0.5	2.71E−03
TP53	Transcription regulator	− 0.4	9.19E−03
HIF1A	Transcription regulator	− 0.5	9.82E−04
MYC	Transcription regulator	− 1.5	4.88E−05
CCND1	Transcription regulator	− 1.6	1.54E−04
SYVN1	Transporter	− 0.1	7.30E−04

## Discussion

Although aging is a widely studied subject and noted as a major risk factor for many chronic diseases, age-related changes in the tear film have not been previously examined in a molecular level using discovery proteomics. Previous research studying tear protein levels during aging have mainly focused on individual, well-known targeted tear proteins alone. Micera et al. [20] identified several pro-inflammatory proteins increasing with age through chip arrays and McGill et al. [10] found a decrease of lysozyme, lactoferrin and IgA and an increase of ceruloplasmin and IgG in tears during aging in their study. In order to obtain more comprehensive image of the protein profiles during aging, we analysed the proteomic expression levels of tear fluid samples among subjects, both male and female, with varying ages using SWATH-MS. The SWATH approach enabled us to perform analyses separately in each individual sample, avoiding this way pooling of the samples. We discovered that, among hundreds of proteins, there is a small subgroup of proteins in tear fluid, which correlated with age and many of these proteins were connected to inflammation, which is known to be increased or at least altered with aging [13, 20, 25]. Majority of the identified proteins had the most notable increase/decrease in expression among subjects aged 60 or over, suggesting that the normal onset for the changes often occurs after this age. Although the identified protein changes may not directly point the specific underlying mechanism that is triggered during aging, these results provide a list of interesting proteins for future tear fluid proteomic studies associated with aging.

Among the proteins, we found age to positively correlate with ALB, ANXA1, DEFA1, LCN2, TF, SCGB2A2, S100A8 and S100A9. All of these proteins have been observed to be increased in the tear fluid proteomics studies examining dry eye disease or other similar inflammatory ocular surface conditions [26–34]. Several of these proteins, most prominently S100A8 and S100A9, are also used as common indicators of ocular surface inflammation [26, 35]. Hence, as also suggested by other tear fluid proteomic studies [20], it would appear that aging does increase the inflammation in the ocular surface and these increased proteins could partially explain why higher age is one of the main risk factors of ocular surface conditions, which are often closely connected to ocular surface inflammation.

Other proteins increasing with age, which could not be directly connected to ocular surface inflammation, included two cytoplasmic actin isoforms (ACTB and ACTG1), GSN, PFN1, CEACAM7 and RARRES1. Of these proteins, CEACAM7 and RARRES1 have not yet been connected to ocular surface condition and while ACTG1 is an interesting protein, it has so far been only connected to treatment effects of dry eye [21]. However, it merits further investigations of its role in ocular surface functions. ACTB has been found to be decreased in the tear fluid of subjects suffering from meibomian gland dysfunction [29], yet it was upregulated in Sjögren’s syndrome [34], which is often considered to be closer to aqueous-deficient dry eye condition. GSN and PFN1 have also been shown to be upregulated particularly in aqueous-deficient dry eye with and without lipid deficiency, but not in lipid-deficient dry eye alone [36]. These previous findings together with our results suggest that the increase of at least some of these proteins would result in increased risk of aqueous-deficient dry eye during aging. However, age-associated, increased risk of meibomian gland-associated lipid deficiency cannot be determined based on these results and instead, e.g. lipidomics could provide more comprehensive results.

Our data analysis also resulted in two proteins with a significantly decreasing expression with increased age: UBA1 and GOLM1. UBA1 has been associated with increased expression in the tear fluid of dry eye patients with both aqueous and lipid deficiency [36]. GOLM1, although there are currently no studies connecting this protein to the ocular surface, has been recently associated with viral infection and its associated immune response [37].

Interestingly, protein-age correlations were in most cases more significant with males, i.e. males’ protein expression increased/decreased more consistently with age. SCGB2A2 was the only protein, which had a somewhat larger correlation coefficient as well as more visible increase for female subjects. However, the overall protein expression directions were not differing between female and male groups, although a previous study by Ananthi et al. [38] identified some upregulated proteins in female tear samples including lipocalin and mammoglobin B precursor, which belongs to the same secretoglobin family as SCGB2A2. Clinical studies have also shown that the dry eye disease, Sjögren’s syndrome and other ocular surface conditions are more common among older women [17, 18], which does suggest that our findings are not associated with increasing prevalence of ocular surface diseases alone but rather normal aging, which appear to be affecting males more significantly. Older women, often suffering from ocular surface disease and its symptoms, are not fully represented in this study, since they are unlikely to participate in ocular surgeries and are in fact excluded in severe cases.

The pathway analysis was performed based on the correlation results originating from complete data, in order to discover general effects of aging. The results showed that while cell survival and organismal growth were decreased, organismal death, inflammation, angiogenesis and immune response-related actions increased. These biological functions display similar results, which have already been identified previously [13, 14, 20, 25]. Several studies have also discussed of the increased immunosenescence, i.e. how the immune system slows down as we age, but also that innate immune system is at the same time incorrectly activated. Therefore, our results with an increase in immune cell chemotaxis and response could refer to the increased innate immune response.

The upstream regulators, which are associated with or affecting protein expression levels observed in the data were also evaluated. Transcription regulator CEBPA, cytokine IL15 and NF-κB complex were the top three activated upstream regulators and of these, NF-κB appears to be the most interesting. This complex is not only connecting to or regulating the other upstream regulators looked at more closely in this study, but various studies and reviews link it to aging, inflammation and dry eye. In fact, NF-κB is considered as a hallmark of aging and master regulator of innate immunity [39, 40]. It has also been identified in rat cornea, conjunctiva and lacrimal gland [41] and a very comprehensive review shows how it links to several ocular surface diseases [42]. Our results therefore comply well with the previous findings and suggest that activation of NF-κB is also present in the aging ocular surface and its tear fluid. Targeting this complex in ocular surface treatment methods could provide good results, especially for elder subjects.

Of the two other activated upstream regulators, CEBPA has been associated to regulation of aging in mouse gene expression as well as fat metabolism [43] and it also inhibits CDK4 and therefore cell proliferation and growth [44]. IL15, which is closely connected to immune system response, has been noted to be increased in the sera of subjects above the age of 95 years [45].

The top three inhibited regulators included transcription regulators CCND1 and MYC as well as CDK4/6 group. In fact, CDK4/6 and CCND1 form together a complex, which regulates cell cycle [46] and has been for that reason covered in various cancer studies. In addition, MYC has also been connected to the CDK4/6 and that way to CCND1 through these cancer studies [47]. Since all three regulators promote growth and regulate cell cycle, it seems feasible that these regulators are inhibited as our results suggest decreased cell survival and organismal growth.

## Conclusions

In conclusion, we discovered several tear fluid proteins, which are significantly affected by increasing age. These proteins are connected to several age-associated functions, e.g. cell death and inflammation, as well as well-known upstream regulators, NF-κB being one of the most interesting of these. In the future tear fluid proteomics studies, the information presented here should motivate researchers to ensure age is properly controlled for in the studies and in addition, the knowledge of the proteins, pathways and upstream regulators can be implemented in the research of dry eye and other ocular surface diseases, which are associated with increased age.

## Additional file

**Additional file 1.** Analysis results of proteins with unadjusted p-value < 0.05.

## Abbreviations

SWATH: sequential window acquisition of all theoretical fragment ion spectra
MS: mass spectrometry
FS-LASIK: femtosecond laser in situ keratomileusis
SDS: sodium dodecyl sulphate
TEAB: triethylammonium bicarbonate buffer
IAA: iodoacetamide
TFA: trifluoroacetic acid
DDA: data-dependent acquisition
LC: liquid chromatography
FDR: false discovery rate
ICC: intraclass correlation
IPA: ingenuity pathway analysis
GO: gene ontology
ACTB: actin, cytoplasmic 1
ACTG1: actin, cytoplasmic 2
ALB: albumin
ANXA1: annexin A1
CEACAM7: carcinoembryonic antigen-related cell adhesion molecule 7
DEFA1: neutrophil defensin 1
GSN: gelsolin
LCN2: neutrophil gelatinase-associated lipocalin
PFN1: profilin-1
RARRES1: retinoic acid receptor responder protein 1
SCGB2A2: mammaglobin-A
TF: serotransferrin
UBA1: ubiquitin-like modifier-activating enzyme 1
GOLM1: Golgi membrane protein 1
NF-κB: nuclear factor kappa-light-chain-enhancer of activated B cells
CEBPA: CCAAT/enhancer binding protein α
IL15: interleukin 15
MYC: Myc proto-oncogene protein
CCND1: cyclin D1
CDK4/6: cyclin-dependent kinase 4 and 6
